# Nuclear import of *Xenopus* egg extract components into cultured cells for reprogramming purposes: a case study on goldfish fin cells

**DOI:** 10.1038/s41598-019-39500-y

**Published:** 2019-02-27

**Authors:** Nathalie Chênais, Thierry Lorca, Nathalie Morin, Brigitte Guillet, Hélène Rime, Pierre-Yves Le Bail, Catherine Labbé

**Affiliations:** 1 INRA, UR1037 LPGP, Fish Physiology and Genomics, Campus de Beaulieu, F-35000 Rennes, France; 2 Centre de Recherche en Biologie Cellulaire de Montpellier, UMR 5237 CNRS, Montpellier, France; 3 Centre de Ressources Biologique Xenope, CNRS Université Rennes 1, Campus de Beaulieu, F-35000 Rennes, France

## Abstract

Reprogramming of cultured cells using *Xenopus* egg extract involves controlling four major steps: plasma membrane permeabilization, egg factors import into the nucleus, membrane resealing, and cell proliferation. Using propidium iodide to assess plasma membrane permeability, we established that 90% of the cultured fin cells were permeabilized by digitonin without any cell losses. We showed that egg extract at metaphase II stage was essential to maintain nuclear import function in the permeabilized cells, as assessed with a fusion GFP protein carrying the nuclear import signal NLS. Moreover, the *Xenopus-*egg-specific Lamin B3 was detected in 87% of the cell nuclei, suggesting that other egg extract reprogramming factors of similar size could successfully enter the nucleus. Lamin B3 labelling was maintained in most cells recovered 24 h after membrane resealing with calcium, and cells successfully resumed cell cycle in culture. In contrast, permeabilized cells that were not treated with egg extract failed to proliferate in culture and died, implying that egg extract provided factor essential to the survival of those cells. To conclude, fish fin cells were successfully primed for treatment with reprogramming factors, and egg extract was shown to play a major role in their survival and recovery after permeabilization.

## Introduction

Egg extract obtained from metaphase-II arrested spawned oocytes consists of a mixture of cytoplasmic and nuclear components, rich in numerous reprogramming factors involved in remodeling zygote chromatin and sustaining early embryo development^1^. Egg extracts have been reported to be valuable media for cellular reprogramming in culture, with the aim of restoring some pluripotency to differentiated cells. The ability of egg extract to remodel the nuclear chromatin of different somatic cell types towards a pluripotent state have been largely explored *in vitro* in mammals: epigenetic profile changes^1–5^ and nuclear chromatin remodeling^3,4,6,7^ have been reported, together with re-expression of silent pluripotent markers such as Oct4, Nanog, Sox2^1,3,5,7,8^ and down-regulation of somatic genes^6^. Most of these studies ultimately intended to improve nuclear transfer outcome, where a donor somatic cell is incorporated into a recipient oocyte and is expected to withstand embryo development. Although the reprogramming success is variable among studies, it was demonstrated that pre-treatment of the donor cell with egg extract led to better blastocyst rate after nuclear transfer in bovine and porcine samples^1,2,8–10^ indicating a beneficial effect of egg extract on the development of the reconstructed embryo. In fish, somatic cell nuclear transfer is a promising method for restoring precious genomic resources from diploid material stored in cryobanks^11^. This would compensate for the fact that fish eggs or embryos cannot be cryopreserved^12^. However, less than 1% fertile adults can be regenerated by this technology^11,13–15^. Because one hypothesis for these low rates is that the donor cell genome is not fully reprogrammed into an embryonic one^16^, a preliminary reprogramming of the donor cell prior to nuclear transfer could also be necessary in these species. To our knowledge, no reprogramming of donor cells in culture has been reported in fish and no information is available on the capacity of cultured fish cells to withstand the biologically demanding steps necessary for such treatments.

The interspecific efficiency of *Xenopus* egg extract to ensure the epigenetic remodeling of somatic cell chromatin in mammals makes it an ideal candidate to test on fish cells. Cellular reprogramming by *Xenopus* egg extracts first requires the plasma membrane to be permeabilized, so that large proteins from the extract can enter the cytoplasm of the cells. Reprogramming factors must then reach the nucleus where they are more likely to interact with chromatin to change the cell expression pattern^2,5,7^. Very often, permeabilization consists in increasing plasma membrane permeability or in creating physical pores in the plasma membrane so that exogenous molecules can cross it passively. Permeabilization methods include electro-permeabilization and permeabilization using pore-forming factors: bacterial toxins such as alpha-toxin or streptolysin O, or pore-forming detergents from plants such as digitonin. These two latter molecules are often preferred because they allow the delivery of large molecules into the cytosol of permeabilized cells^3,4,7–10^: with digitonin and streptolysin O, passive incorporation of up to 100 kDa proteins was reported^17,18^. Because digitonin is less toxic than streptolysin O and operates faster, digitonin is more frequently used in cell culture^19^. Furthermore, the strong affinity of digitonin for cholesterol allows only the cholesterol-rich plasma membrane to be permeabilized while the membranes of nuclei, mitochondria and other intracellular organelles are not altered by digitonin^20,21^. Lastly, digitonin-permeabilization is thought to be reversible, as the resealing of the plasma membrane and resumption of cell culture has been reported for several mammalian cell types^7,8,22^. However, one problem with trying to reprogram cultured cells after permeabilization is that the pores thus created also allow the loss of cytosolic components that may be necessary for signal-transduction pathways, metabolic activity and other cellular functions in the cells, such as nuclear import. Factors important for cell survival and transport of molecules to the nucleus may therefore be lost^20,21^. In all, before any study on the reprogramming of cultured cells by egg extract can be conducted, each stage of the treatment process must be validated, namely plasma membrane permeabilization, maintenance of nuclear import, plasma membrane resealing, and cell growth resumption in culture. Variability of the cell response at each stage must also be carefully assessed.

In this work, we studied the response of goldfish fin cells to treatment with *Xenopus* egg extracts, with the objective of validating a system for later use in chromatin reprogramming. We first sought the best permeabilization conditions using digitonin, and evaluated cell permeabilization yields with non-permeant markers of different molecular size. Maintenance of the cell nuclear import capacity of the permeabilized cells was also assessed by tracking the nuclear import of a fusion protein carrying a nuclear localization signal (NLS). Finally, we examined the treated cells recovery, viability in the presence of calcium, a pore-resealing molecule, and ability to proliferate in culture. The overall objective of the work was to provide a step-by-step demonstration of the capacity of fish fin cells to be successfully prepared for cell reprogramming using *Xenopus* egg extracts.

## Results

### Permeabilization of the fin cell plasma membrane by digitonin

**Figure 1:**
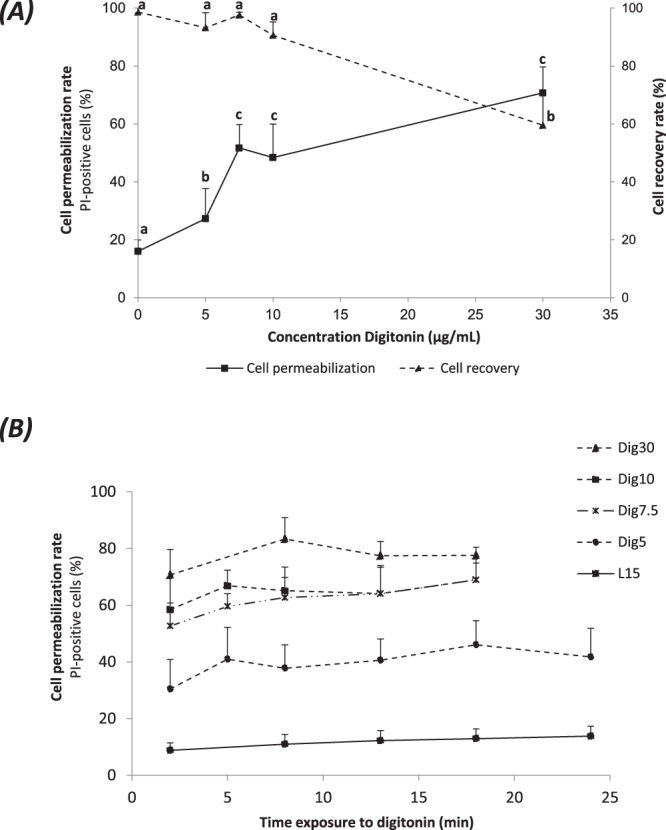
Digitonin-induced permeabilization of fin cells in suspension. Mesenchymal fin cells were tested for their ability to respond to various digitonin treatments. (**A**) Permeabilization rates and cell recovery rates after 2 min incubation. (**B**) Response over time of digitonin-induced permeabilization (2 to 24 min). Cells were incubated at digitonin concentrations ranging from 5 to 30 µg/mL and labelled with the non membrane permeant propidium iodide (PI). Cell permeabilization rates are expressed as a percentage of PI-positive cells to the total cell number in the sample. Cell recovery rates are expressed as a percentage of the cell number after digitonin treatment to the initial cell number before treatment. Bars represent means ± SD (n = 3 to 13 independent fin cell suspension). In (A), different letters indicate significant differences (p < 0.01) for a given concentration.

We screened a range of digitonin concentrations over time at 4 °C to find the best compromise between plasma membrane permeabilization and cell survival. To facilitate assessment of permeabilization success, this screening was first performed on cells in suspension. Propidium iodide (PI) was used as a reporter of plasma membrane permeabilization because of its inability to permeate intact plasma membranes. At the shortest treatment duration (2 min), the permeabilization rates increased with increasing digitonin concentration, as observed from the increase in PI-positive cells (Fig. 1A). The percentage of PI-positive cells in the controls without digitonin indicates that only a small fraction of the cells without any treatment had an altered plasma membrane. More than 70% of the cells were PI-positive at 30 µg/mL digitonin, although the overall efficiency of this concentration was not significantly different from 10 and 7.5 µg/mL digitonin. Even the lowest dose, 5 µg/mL digitonin, induced some significant membrane permeabilization. Digitonin toxicity remained low as indicated by the high recovery rates (number of cells counted at the end of the digitonin treatment) observed from 0 to 10 µg/mL digitonin. Even at the highest digitonin concentration, 30 µg/mL, cell recovery was still 60% (Fig. 1A). A longer incubation time did not increase the permeabilization rate (Fig. 1B). This suggests that a 2 min exposure to digitonin is sufficient to realize most of the digitonin permeabilization potential at a given concentration. The shortest exposure time, of 2 min, was thus selected for subsequent experiments.

**Figure 2:**
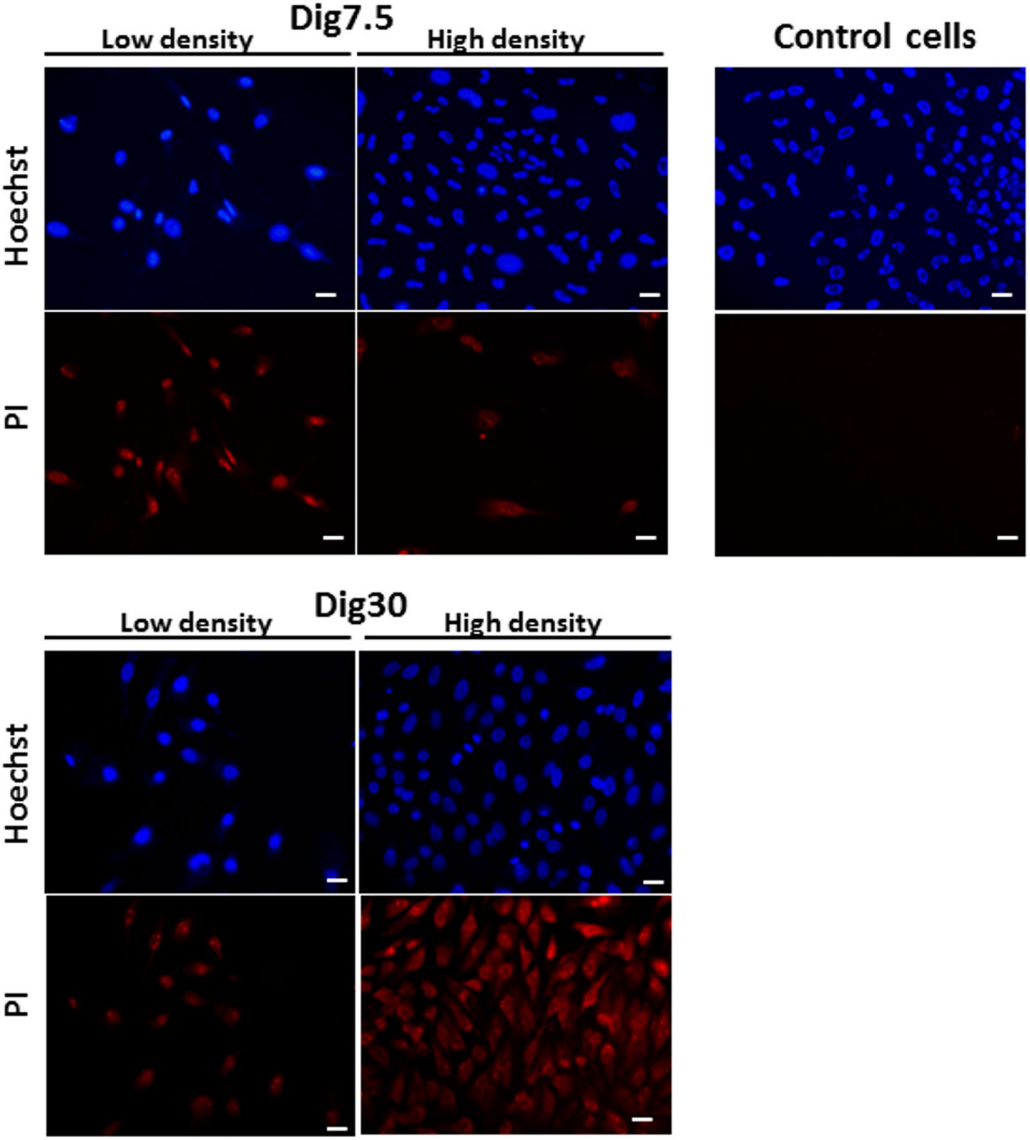
Digitonin-induced permeabilization of adherent fin cells in relation to cell density. Cells were permeabilized by digitonin at 7.5 µg/mL (Dig7.5) and 30 µg/mL (Dig30). After 2 min exposure at 4 °C, cells were labelled with Hoechst 33243 (upper picture) and the non membrane permeant propidium iodide (PI) (lower pictures). The permeabilized cells were identified by a red PI fluorescence observed in low and high density areas. Note that no red fluorescence was detected in non-permeabilized cells (Control cells) included in this experiment. These pictures are representative of seven independent replicates. Scale bar = 20 µm.

Among the three most efficient digitonin concentrations that were tested on cell suspension, we applied the lowest and the highest dose to adherent cells. These cells consist in a population of homogeneous cells containing almost only mesenchymal cells (>95%) with some melanophores remaining present (1%). This homogeneity resulted from the two-steps plating procedure that was set up in this study. The population obtained with this faster new method was similar to the one described previously in explant culture^23^. The objective of using adherent cells was to validate the permeabilization conditions in a culture system that would later be the most suitable for treating the cells with egg extract. At 7.5 µg/mL digitonin, the cell permeabilization rate was influenced by cell density (Fig. 2). In low density areas, the well spread adherent cells were almost all PI-positive whereas in high density areas, only few were PI-positive. This lower permeabilization rate observed for cells at high density could be explained by a reduced membrane exposure to digitonin because of their reduced cell surface, leading to a lower PI penetration. The overall permeabilization rate at 7.5 µg/mL digitonin was about 50% for adherent cells (n = 7 independent cultures), a value that was similar to that of the cells in suspension. For adherent cells, the permeabilization pattern was more thorough and homogenous at the highest digitonin dose. Approximatively 90–95% of the adherent cells incubated with 30 µg/mL digitonin were PI-positive regardless of cellular density. This is even higher than the 70% rate obtained with cells in suspension and suggests a higher sensitivity of adherent cells to 30 µg/mL digitonin compared with round shaped cells in suspension. Interestingly, at this concentration, no cell detachment from the culture dish or nuclei DNA fragmentation were observed (Fig. 2). This observation is at odds with the mild toxicity that was observed with cell suspensions at the same digitonin concentration. This highest 30 µg/mL dose was used for further experiments.

**Figure 3:**
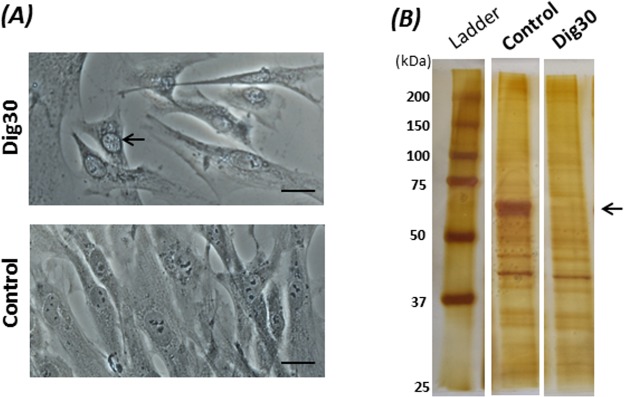
Cell morphology (**A**) and loss of cytoplasmic proteins (**B**) after digitonin-induced permeabilization. The cells were permeabilized for 2 min with 30 µg/mL digitonin at 4 °C. (**A**) The morphology of permeabilized (Dig30) and non-permeabilized (Control) cells was observed by phase-contrast microscopy. Note the nuclear envelope that was more contrasted (arrow) in permeabilized cells than in control cells. Scale bar = 20 µm. (**B**) Digitonin-permeabilized (Dig30) and non-permeabilized (Control) cells were lysed with RIPA buffer added to the adherent cells. Protein lysates were submitted to SDS-PAGE then silver stained. The loss of a protein band at about 60 kDa was observed (arrow) in the permeabilized cells.

The morphology of the adherent cells permeabilized with 30 µg/mL digitonin was no different from that of the non-permeabilized control cells, except that the nuclear envelope often appeared more contrasted (Fig. 3A). Such a change in nucleus appearance provides a visual test that could validate that some sort of digitonin-induced cellular response has taken place. In contrast to the overall morphological stability, changes in protein contents were observed in the permeabilized cells (Fig. 3B). The most visible loss of proteins was observed at the major bands around 60 kDa. This likely indicates a release of cytoplasmic soluble proteins from the permeabilized cells.

### Penetration of large molecules into the cytoplasm of permeabilized adherent cells

**Figure 4:**
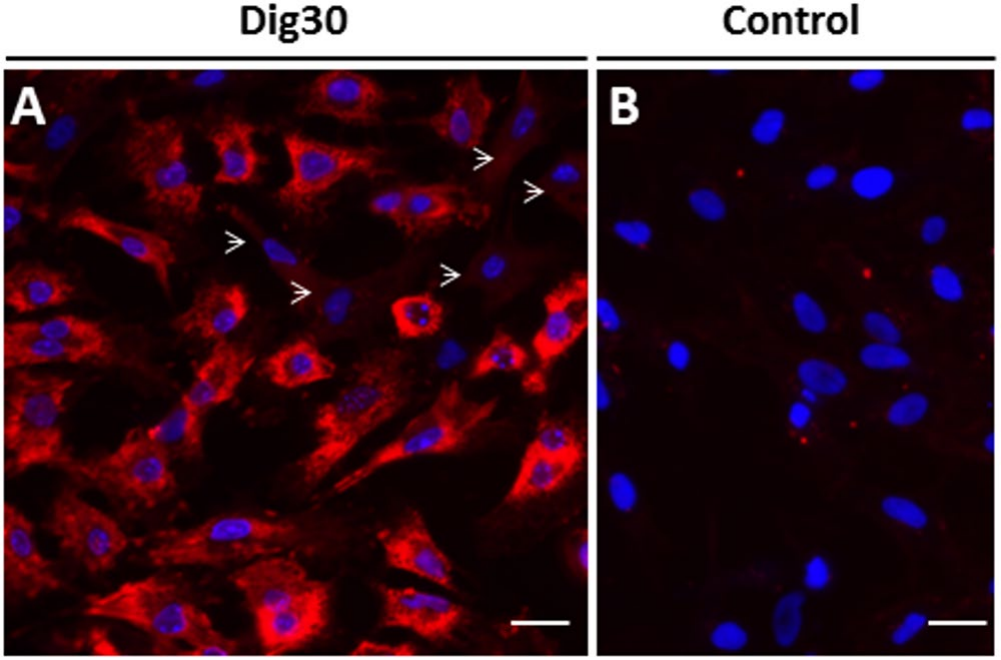
Labelling of permeabilized cells with a large 70 kDa dextran. Cells permeabilized with 30 µg/mL digitonin (Dig30, **A**) and non-permeabilized cells (Control, **B**) were labelled with Texas red-conjugated 70 kDa dextran. The red fluorescence was present in the cytoplasm of permeabilized cells and excluded from nuclei. Most permeabilized cells were strongly labelled, some cells showed lower fluorescence pattern (arrowheads) and only few cells contained no fluorescence (not shown). No fluorescence was detected in the non-permeabilized cells. These pictures are representative of three independent replicates. Scale bar = 20 µm.

The permeabilization obtained in the experiments above was validated by the penetration of small PI molecules (0.648 kDa), and the release of cytosolic proteins of about 60 kDa. In order to test whether the permeabilization treatment was suitable for incorporation of larger molecules that may be required for reprogramming, Texas red-conjugated 70 kDa dextran was added after permeabilization with 30 µg/mL digitonin. We observed that a high proportion of permeabilized cells showed Texas red fluorescence localized in the cytoplasm (Fig. 4). The labelling was absent from the nuclei, and this indicates that the nuclear envelope remained intact during the permeabilization process. The intensity of cytoplasmic fluorescence varied among cells, suggesting some variability in the penetration efficiency of large molecules through the membrane pores (Fig. 4). Without digitonin, no dextran labelling was observed in the cell cytoplasm, even after a much longer incubation time (Fig. 4B). Thus, no dextran was incorporated by endocytosis, and the dextran labelling was solely due to cell permeabilization. Taken together, these results demonstrate that the permeabilization conditions with 30 µg/mL digitonin specifically affected the cell plasma membrane without alteration of the nuclear envelope and that digitonin-induced pores were large enough to allow the penetration of molecules of at least 70 kDa.

### Nuclear import ability of the permeabilized cells

**Figure 5:**
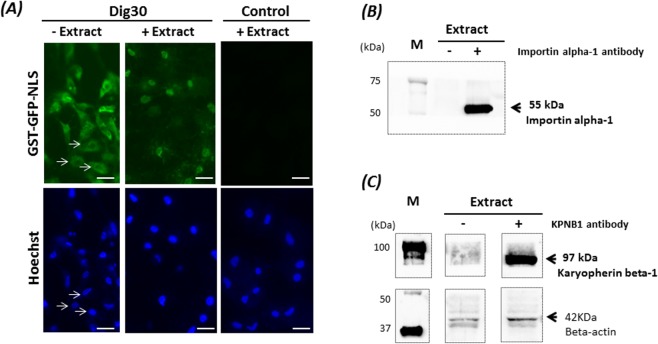
Nuclear import of the fusion protein GST-GFP-NLS in the permeabilized cells. Cells permeabilized with 30 µg/mL digitonin (Dig30) and non-permeabilized cells (Control) were incubated with GST-GFP-NLS in the absence (−Extract) or presence (+Extract) of *Xenopus* egg extract supplemented with ATP for 1 h at 25 °C. The incorporation of GST-GFP-NLS was assessed by green fluorescence. Cell nuclei were counterstained with Hoechst 33243. Note that without egg extract, the nuclei are not labelled (arrows). Due to light scattering of the fluorescence in the cytoplasm, the nuclei appear smaller than they are. The egg extract restored nuclear import in the permeabilized cells. These pictures are representative of five independent replicates. Scale bar = 20 µm. Detection of importin alpha-1 (55 kDa) (**B**) and Karyopherin *beta*-1 (KPNB1–97 kDa) (**C**) in *Xenopus* egg extract by western blot in the presence (+) or in absence (−) of the anti-*Xenopus* importin alpha-1 antibody (clone 15) and the anti-rat KPNB1 antibody (KPNB1-clone 23). Beta actin was used as a loading control. M: size markers. The cropped blots came from the same gels and were analyzed with the same exposure times (B:1 sec; C:1 min).

Because chromatin is an important target for cellular reprogramming, it is important that permeabilized cells maintain their ability to sustain the nuclear import of large cytoplasmic proteins. It can be seen from Fig. 4 that the nuclei were not permeable to the exogenous dextran, which indicates the maintenance of the nuclear barrier in the permeabilized cells. Transport of cytoplasmic macromolecules to the nucleus is regulated by nuclear pore complexes. Only molecules carrying a specific signal, such as the nuclear localization signal (NLS), will be transported. The nuclear import ability of the permeabilized cells was thus tested at 25 °C with a fusion GFP protein containing the NLS group (GST-GFP-NLS). We showed that the nuclear import of the GST-GFP-NLS was not functional in the permeabilized cells. Indeed, the fluorescence of GST-GFP-NLS was restricted to the cytoplasm of the permeabilized cells (Fig. 5A). However, when the permeabilized cells were incubated with *Xenopus* egg extract in the presence of the GST-GFP-NLS, fluorescence was observed in the nuclei, indicating that the nuclear import function was functional. This labelling was observed in 87 ± 4% of treated cells. In addition, we showed that the extract contained two members of the importin family: importin alpha-1 and karyopherin beta-1 (also known as importin beta-1), of expected sizes of 55 and 97 kDa, respectively (Fig. 5B,C). No nuclear fluorescence was detected in the permeabilized cells if the import assay was carried out at 4 °C, even in the presence of *Xenopus* egg extract, indicating that the import is temperature dependent (data not shown). Taken together, these results indicate that the *Xenopus* egg extract and the temperature of 25 °C are essential to the success of nuclear import of NLS-proteins in the fin permeabilized cells.

**Figure 6:**
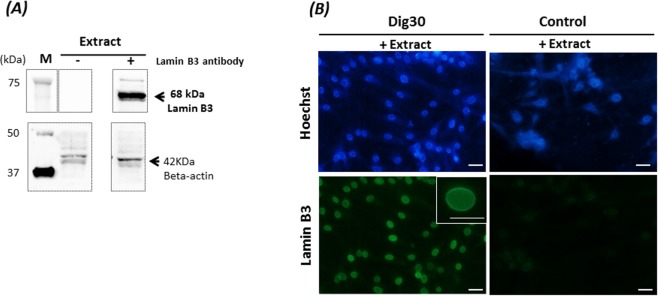
Incorporation of Lamin B3 from *Xenopus* eggs into the nuclei of permeabilized cells. (**A**) Detection of Lamin B3 (68 kDa) in *Xenopus* egg extract (Extract) by western blot in the presence (+) or absence (−) of anti-*Xenopus* Lamin B3. Beta actin was used as a loading control. M: size markers. The cropped blots came from the same gel and were analyzed with the same exposure times (Lamin B3:2 sec; beta actin: 1 min). (**B**) Nuclear import of Lamin B3 detected by immunofluorescence in permeabilized (Dig30) and non-permeabilized (Control) cells incubated for 60 min in the presence (+Extract) of *Xenopus* egg extract under energy supplementation at 25 °C. Lamin B3-positive cells presenting a detectable signal (from weak to strong staining) were observed. A high proportion of nuclei were labelled. Inset: magnification of one nucleus showing a strong labelling of the nuclear lamina. These pictures are representative of five independent replicates. Scale bar = 20 µm.

As a final validation of the ability of permeabilized cells to import exogenous molecules into their nuclei, our next step was to establish whether some proteins from the *Xenopus* egg extract could be found in the permeabilized cell nuclei. Lamin B3 (68 kDa) is the major *Xenopus* egg lamin that contains a classic NLS^24^. This maternal nuclear protein contributes to the maintenance of the structural integrity of the nuclear envelope during early embryonic development^25^ and is not found in somatic tissues^26^. By a western blot we confirmed the presence of this *Xenopus* egg-specific Lamin B3 in all extract batches that were used to treat the permeabilized cells (Fig. 6A). When permeabilized cells were incubated with *Xenopus* egg extract, a high proportion of nuclei (87 ± 6%) were positive for this exogenous protein (Fig. 6B). Interestingly, this proportion was the same as the one after GFP-NLS protein import. The strong and ring-shaped labelling observed on the nuclei suggests that the integrity of the nuclear lamina was maintained (Fig. 6B inset). No labelling was found when the cells incubated with the *Xenopus* egg extract had not been permeabilized beforehand. The presence of *Xenopus* Lamin B3_NLS into nuclei of permeabilized cells after egg extract treatment suggests that other NLS proteins in *Xenopus* extract, such as reprogramming factors, could be transported into the nuclei of the permeabilized fin cells.

A side observation about the beneficial effect of egg extract on permeabilized cells is that nucleus integrity was maintained in cells during incubation with egg extract whereas some nucleus fragmentation was observed in several cells incubated without egg extract (Supplementary Fig. S1). Additionally, right after addition of the egg extract, the nuclei adopted a very contrasted and white pattern with the nucleolus becoming strongly visible, while the cell borders were no longer visible (Supplementary Fig. S2). All the nuclei with such pattern were NLS-labelled.

### Resealing of plasma membrane pores and ability of the treated cells to proliferate in culture

**Figure 7:**
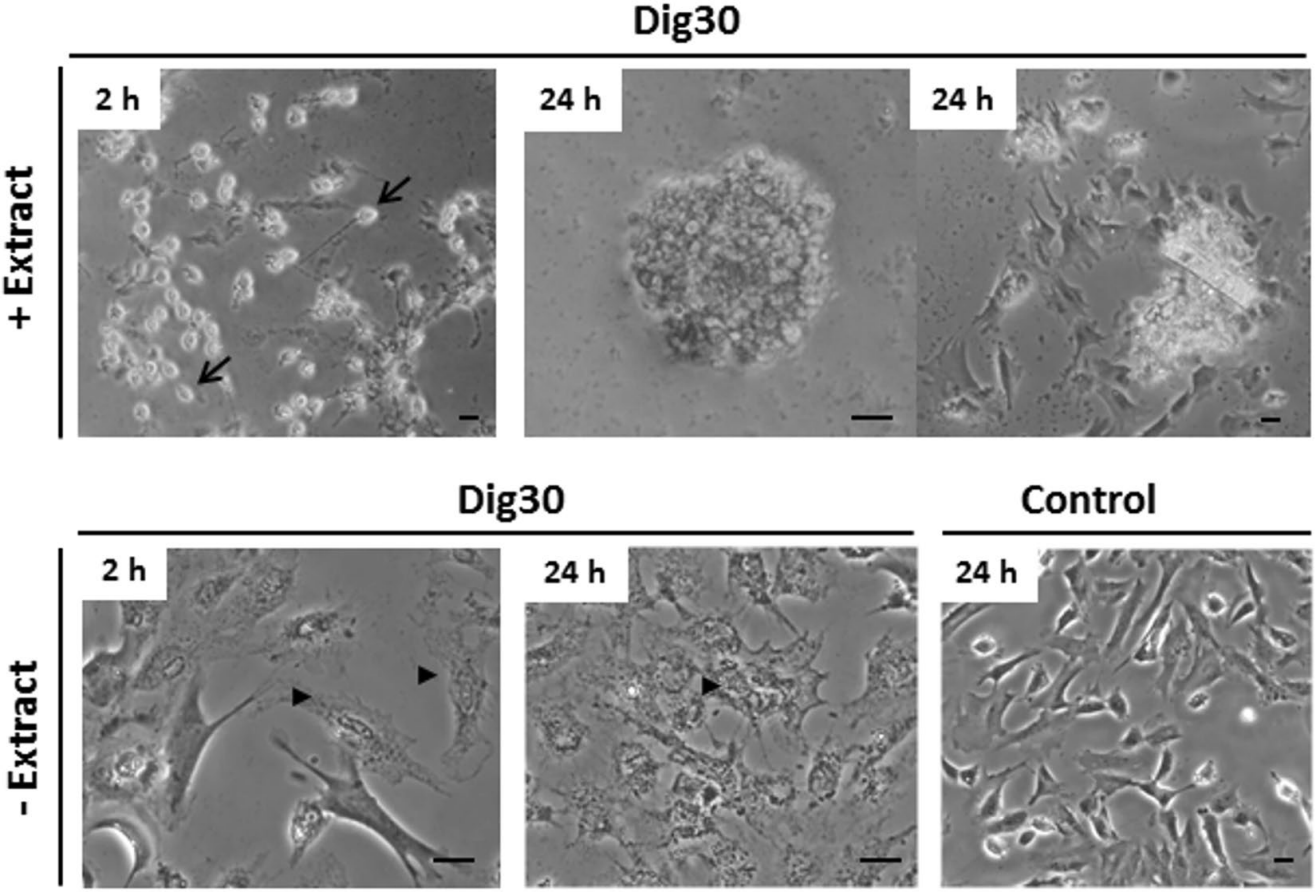
Morphology of the permeabilized cells over the time post-resealing. Permeabilized cells (Dig30) were treated with *Xenopus* egg extract (+Extract) or left without (−Extract) for 1 h at 25 °C. Cells were then incubated in the resealing medium for 2 h. Permeabilized cell behavior was assessed by phase contrast microscopy after the 2 h resealing and 24 h post-resealing. The permeabilized cells that were egg-extract treated exhibited a round and refracting morphology (arrows) that progressively spread. By contrast, the permeabilized cells that were not exposed to the *Xenopus* egg extract did not survive in the resealing medium (arrow heads). These pictures are representative of six independent replicates. Non-permeabilized cells incubated in culture medium were included as control cells. Scale bar = 20 µm.

After cell permeabilization and exposure to *Xenopus* egg extract, resealing was triggered with calcium and the cell behavior was investigated during this resealing phase. During the first two hours of resealing, the cell monolayer adopted a very specific pattern (Fig. 7). We observed an upheaving of many cells which became round and light refracting while remaining attached to the culture dish. However, this population accounted for only 10 to 30% of the initial cell population. Indeed cell attachment was very fragile at this stage. Extract removing and resealing medium addition disturbed the cell layer and induced massive cell loss. Attachment of these round cells to the culture plate was too fragile at this stage to allow the PI incorporation study, so the plasma membrane resealing was not strictly assessed at this time. Twenty four hours post-resealing, we observed round cell clusters that progressively spread on the culture dish, or well-spread cells, or both (Fig. 7). We also demonstrated the absence of PI incorporation in the nuclei of the extract-treated permeabilized cells (Supplementary Fig. S3), indicating a successful restoration of plasma membrane integrity in the 24 h resealed cells. This behavior was specific to cells exposed to the extract. None of the control cells survived more than few hours. Indeed, most of those permeabilized cells that were not treated with the *Xenopus* egg extract remained flat and barely visible (Fig. 7). They displayed marked alterations in their morphology, with a granular cytoplasm (Fig. 7). This was associated with strong nucleus fragmentation (data not shown) during the resealing time, a majority of PI labelled cells at 24 h, and ultimately cell death. An energy supplementation of the resealing medium did not improve the quality of these permeabilized cells (data not shown).

**Figure 8:**
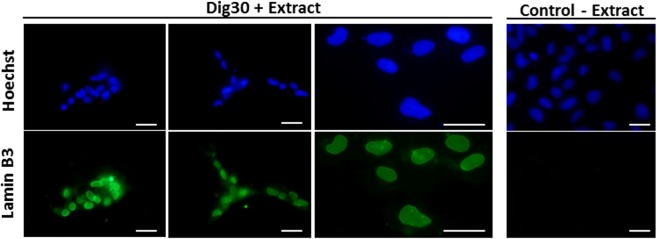
Detection of *Xenopus* Lamin B3 in treated cells 24 h post-resealing. Permeabilized cells were treated with *Xenopus* egg extract (Dig30 + Extract) for 1 h at 25 °C and incubated in resealing medium for 2 h. At 24 h post-resealing, Lamin B3 was detected by immunofluorescence in the nuclei of recovered cells. Nuclei were counterstained with Hoechst 33243. Several representative fields are shown including an enlarged view. Non-permeabilized cells were incubated in absence of *Xenopus* egg extract (Control – Extract). These pictures are representative of three independent replicates. Scale bar = 20 µm.

In order to ensure that the cells recovered at 24 h originated from cells that had been permeabilized and exposed to *Xenopus* egg factors, the presence of Lamin B3 was checked for after the resealing phase. We observed at 24 h that 74 ± 20% of recovered treated cells contained *Xenopus*-egg Lamin B3 in their nuclei and did not show any nucleus fragmentation (Fig. 8). Furthermore, 78 ± 11% of egg-extract treated cells were BrdU-labelled. This proliferation ability of the treated cells was not significantly different from the control (non-permeabilized) cells (87 ± 3%) indicating that treated cells were able to resume proliferation in normal culture conditions.

## Discussion

In this work, we demonstrated that fin somatic cells could be permeabilized by digitonin treatment and that this permeabilization allowed the penetration of exogenous proteins from *Xenopus* egg extract. We also demonstrated that under favorable conditions of temperature and energy, *Xenopus* egg extract restored the cellular nuclear import of NLS proteins that had been impaired in the permeabilized cells. Lastly, only the permeabilized cells treated with egg extract were able to proliferate in culture.

### Permeabilization of fin cells in culture

We report that the shortest digitonin exposure time and highest digitonin concentration tested yielded the best permeabilization rates in cultured fin cells. Such a fast action of digitonin in generating plasma membrane pores has also been reported in different cultured cell types in bovine, ovine and porcine species^2,7,9,27^, where the same high digitonin concentration (30 µg/mL) also yielded the best permeabilization rates^6,7,9^. However, this digitonin concentration was always associated with significant cell detachment and death^7,9^, and this led the authors to select lower concentrations for routine cell treatment. That fish fin cell viability in adherent cell culture was not affected by high digitonin concentration indicates that our model is less sensitive to digitonin toxicity. Because it is known that the action of digitonin is mediated by plasma membrane cholesterol, differences in digitonin toxicity could be related to differences in the cholesterol content of the plasma membranes according to the cell type and species. Such interspecies variability in cholesterol was reported in sperm for instance^28^. It also cannot be excluded that cell morphology may influence sensitivity to digitonin, as we showed that round cells in suspension were more affected than spread adherent cells. These specificities support the work of Miyamoto *et al*.^7^, where the authors proposed that digitonin doses should be set specifically for each new cell type. Additionally, our finding about the nuclei being more contrasted after digitonin treatment was also reported by Adam *et al*.^17^ in HeLa cells. This provides a useful morphological marker of the cells that were successfully permeabilized. We infer that membrane permeabilization may have induced some cytoplasm exiting, causing the cell to flatten while the nucleus remains bulging.

Being able to use high digitonin concentration on fin cells proved to be favorable to the penetration of macromolecules above 70 kDa. In their work on porcine cells^9^, Liu *et al*. had to reduce the digitonin treatment to 7.5 µg/mL, and observed that although 60% of the cells were still PI-positive and thus permeabilized, less than 10% were labelled with 70 kDa Dextran. We infer that the entrance of large exogenous molecules into the cells was enhanced in our case thanks to digitonin concentrations that were higher than in the porcine cells, leading to larger pores.

### Nuclear import in permeabilized cells

The successful permeabilization of plasma membranes allowing the transfer of high molecular weight molecules does not presuppose the maintenance of cellular functionality. We focused more specifically on the functionality of one of the nuclear import pathways. The best studied import pathway is the import of proteins with a classic NLS sequence. This import is mediated by proteins belonging to the karyopherin family, namely importin alpha (about 60 kDa) and importin beta (97 kDa), and regulated by the GTPase Ran. The import process is initially triggered by the binding of importin alpha to the protein-NLS sequence. Then, importin beta binds to the importin alpha/protein-NLS complex to ensure the transport and anchorage of this trio to the nuclear pore complex. The translocation of the trio inside the nucleus requires the binding of importin beta to the GTPase Ran, leading ultimately to the release of the NLS protein into the nucleus compartment^29^. In our digitonin permeabilized cell system, the nuclear import of the recombinant protein tagged with NLS was unsuccessful despite energy supply and favorable temperature. It could be hypothesized that fish importins cannot support the GST-GFP-NLS import but this is unlikely because of the ubiquitous NLS signal that was used^17^. Indeed, this protein sequence has been shown to be supported by a very wide range of importins, from E. coli and S. cerevisiae to HeLa and rabbit cells^17,30^. The most likely hypothesis is that endogenous proteins belonging to the nuclear import pathway were lost through the large pores of the plasma membrane as already shown in digitonin-permeabilized HeLa cells^17^. Although the lack of antibodies against goldfish importins prevented us to demonstrate this hypothesis, many cellular proteins in the range of 60 kDa were no longer found in the fin permeabilized cells. Preservation of the nuclear envelope during the permeabilization process suggests that the protein losses would originate exclusively from the cytoplasm. Cytoplasmic protein release was also reported during the permeabilization of mammalian cells treated with digitonin. In rat hepatocytes and astrocytes or in Hela cells, 18 to 30% of the total proteins were lost^20,21,31^ and several authors also reported the loss of the nuclear import function^21,32,33^. Although we did not characterize the specific proteins lost, we suppose that the endogenous importin alpha-1 of 60 kDa left the cell via the plasma membrane pores. Loss of endogenous importin beta-1 (of bigger size than importin alpha-1) cannot be excluded either, even if this was not clearly demonstrated in our study. Interestingly for our objective, nuclear import was functional in the presence of *Xenopus* egg extract. This suggests that the cytosolic factors contained in the *Xenopus* egg extract and involved in nuclear import, such as importin-alpha-1 and importin beta-1 shown to be present in our egg extract, were interchangeable with their fish counterparts. This hypothesis is supported by the results of Kose *et al*.^33^, who showed that providing both recombinant alpha and beta importins to digitonin-permeabilized Hela cells in the presence of the additional actor Ran-GDP and ATP restored nuclear import capacity. Beyond the efficiency of heterologous proteins for maintenance of nuclear import in fish cells, we demonstrated that heterologous proteins carrying the NLS were successfully transported into the fish cell nucleus, as shown by the detection of Lamin B3, a major *Xenopus* egg lamin^26^, in the nuclei of our permeabilized cells.

### Permeabilized cell recovery: plasma membrane healing and cell proliferation

The triggering of plasma membrane repair process requires addition of extracellular calcium^18,34,35^ although repair mechanisms differ according the type of damage. The repair of pore-containing plasma membrane areas triggers a cascade of events leading to membrane endocytosis that is strongly dependent on cellular ATP and calcium^35^. In our system, we demonstrated that calcium was not sufficient to trigger complete plasma membrane recovery, because only cells that had been previously exposed to *Xenopus* egg extract were able to recover and proliferate. It cannot be ruled out in our system that membrane repair would involve vesicles derived from the Golgi apparatus in egg extracts that would fuse with permeabilized areas of plasma membrane, as observed in case of repair of large mechanical wounds in which these vesicles act as patches after exocytosis^36^. Indeed, *Xenopus* egg extracts were prepared at low centrifugation, allowing the ooplasmic membrane vesicles to be maintained in the extract. This provided membrane vesicles that may have contributed to the healing of digitonin-induced alteration of the plasma membrane. But cell recovery may not be relying only on membrane repair. In the absence of *Xenopus* egg extract, factors essential to cell survival were probably lost in our conditions of permeabilization. The releasing of endogenous stores of ATP, as demonstrated in the SLO-permeabilization system in mammals^18^ is one important component involved here. However, the provision of energy substrates such as ATP and creatine phosphate were not enough to sustain cell viability in our system when no egg extract was present. It is likely that our cells may have lost important metabolic enzymes such as lactate dehydrogenase, as reported in digitonin-permeabilized murine cells^37^, where up to 65% of total lactate dehydrogenase content was lost. In our system, whatever the missing molecules, they could be provided by the egg extract.

To conclude, this study thoroughly explored the cellular response of cultured fish fin cells to permeabilization treatment with digitonin. We demonstrated also that the egg extract provided cellular components that are vital for the maintenance of the permeabilized cell function, including nuclear import, membrane recovery and cell cycle resuming. Above all, the system ensured that a high proportion of recovered cells whose nuclei were labelled with egg extract markers survived in culture. This provides a solid basis for further studies in cellular reprogramming because all steps necessary to ensure that reprogramming factors can access cell chromatin were validated. This first demonstration in fish also illustrates the interest of fish fin cells for reprogramming studies, thanks to their excellent response to digitonin-induced permeabilization and egg extract treatment.

## Methods

### Fin cell culture

Two-year-old goldfish (*Carassius auratus*) of 40 g mean weight were obtained from outdoor ponds at the INRA U3E experimental facility (Rennes, France) and maintained in the INRA LPGP recycled water facility at 14 °C. After euthanasia according to animal welfare guidelines and under the French registration authorization n° 78–25 (N. Chênais), caudal fins were collected and dissociated with collagenase at room temperature. Fins were obtained from adult goldfish and dissociated with collagenase. Cells were plated in 6-well plates in growth L15 culture medium (detailed procedure in Supplementary Method 1). After 24 hours, the faster adhering epithelial cells were discarded and the supernatant, enriched with the slower adhering mesenchymal cells was collected. These cells have previously been shown to be the most suitable for nuclear transfer^11,23^. After filtration and washing, the cells were seeded at 0.25.10^6^ cells/well in 24 well plates on 1.3 cm^2^ glass coverslips coated with 10 µg/mL human fibronectin (Sigma F0556) and cultured for 2 days (about 60–70% confluence). This intermediate density is important as we observed a lower efficiency of digitonin in plasma membrane permeabilization when cells were packed in tight clusters.

### Preparation of *Xenopus laevis* egg extract and western blot analysis

The procedures for egg extract preparation and western blotting are described in detail in Supplementary Method 1. Briefly, unfertilized eggs were crushed at 10,600 × g for 20 min at 4 °C. The middle layer containing the cytoplasmic protein fraction was collected and supplemented by protease inhibitors, creatine phosphate (20 mM) and cytochalasin B (75 µg/mL). The extract was then clarified at 10,600 × g for 20 min at 4 °C and the supernatant was stored at −80 °C. After western blotting of the egg extract, the membranes were incubated overnight at 4 °C with rabbit polyclonal *Xenopus* anti-Lamin B3 (1:10 000, a gift from N. Morin, France), *Xenopus* anti-importin alpha1 (1:5000, a gift from K. Weiss, Switzerland), mouse monoclonal rat anti-karyopherin β1 (1:1000 KPNB1, Antibodies-online, clone 23), and anti-beta actin (1:5000, Sigma, clone AC15). Immunolabelling was revealed with Uptima Uptilight HRP Chemiluminescent Substrate (Uptima-Interchim 58372B).

### Digitonin permeabilization of the cultured fin cells

A large range of digitonin concentrations were screened at several incubation times on the cells recovered from the supernatant and adjusted at 2.10^6^ cells/mL in permeabilization buffer (110 mM potassium acetate, 5 mM sodium acetate, 2 mM magnesium acetate, 1 mM EGTA, 2 mM DTT, aprotinin and leupeptin at 1 µg/mL each and 20 mM Hepes pH 7.3). Cells from 13 independent cultures were tested. Digitonin (Calbiochem 300410) was added to aliquots of 100 µL of cell suspension (containing 0.2 10^6^ cells) at 0, 5, 7.5, 10 and 30 µg/mL. After different incubation times at 4 °C (from 2 to 24 min) a cell fraction was collected, labelled with 0.12 µm propidium iodide (PI), and the percentage of cells with permeabilized plasma membranes (PI-positive cells) assessed by flow cytometry (MACSQuant, Miltenyi Biotec). The toxicity of each digitonin treatment was assessed from the cell losses during permeabilization.

The permeabilization procedure was applied to adherent mesenchymal cells in culture. Cells cultured on coverslips were rinsed once with the permeabilization buffer and permeabilized at 4 °C with digitonin according to the chosen treatment (dose and exposure time). Permeabilization was then stopped by adding an excess of cold permeabilization buffer. Coverslips were collected, inverted on a 40 µl droplet of 0.12 µM PI and 10 µg/mL Hoechst 33243 in PBS. The presence of PI-positive cells was monitored over time by fluorescence microscopy (Eclipse 90i Nikon microscope). We checked that PI was not incorporated in non-permeabilized cells after 25 min incubation. The permeabilization rate was expressed as a percentage of the PI-positive cells on the total cell number counterstained with Hoechst. An average of 300 to 500 cells was counted per condition. The permeabilization rates of adherent cells were obtained from seven independent cultures.

Protein losses from permeabilized cells were assessed after permeabilization. The permeabilization buffer containing the material released from the cells was discarded. The adherent cells were covered with RIPA lysis Buffer (50 mM TRIS pH 8, 1 mM EDTA, 0.5 mM EGTA, 1% NP-40, 0.5% sodium deoxyclolate, 0.1% SDS, 150 mM NaCl) supplemented with 5 mM NaF, 1 mM NaVO_4_, and protease inhibitor cocktail (Roche, 11873580001) for 10 min on ice. The cell lysate was then centrifuged at 4 °C 14,000 × g for 15 min. The supernatant containing all the soluble material was mixed with Laemmli buffer 2x (1 v: 1 v). After denaturation, proteins were separated by SDS-polyacrylamide gel electrophoresis and silver stained.

### Labelling of the permeabilized cells with large molecules

After permeabilization, cells were incubated with 50 µg/mL Texas red-conjugated 70 kDa dextran (lysine fixable, Fisher Scientific, D1864) in growth L15 culture medium for 30 min at 25 °C. Coverslips were subsequently mounted in PBS containing 50% glycerol. The penetration of this probe into the cytoplasm of the cells through membrane pores was assessed by fluorescence microscopy.

For the nuclear import study, permeabilized cells and non-permeabilized controls were incubated for 1 h at 25 °C with 2.4 µl Glutathione-S-Transferase Green Fluorescent Protein fused with NLS (GST-GFP-NLS 1 µM, a gift from N. Morin) with 90 µL L15 culture medium or *Xenopus* egg extract supplemented with 2.5 mM ATP. The ubiquitous NLS sequence chosen here was CGGGPKKKRKVED. GST (210 amino acids) is 25 kDa and GFP (238 amino acids) 27 kDa. In all the protein is about 54 kDa for 461 amino acids. Cells were washed with PBS, fixed with 4% PFA for 5 min at RT, and counterstained with 10 µg/mL Hoechst 33243. The nuclear import of GST-GFP-NLS protein was assessed by fluorescent microscopy. The number of cells presenting nuclear GFP labelling was counted in three independent experiments (530 counted cells), after counterstaining with 10 µg/mL Hoechst 33243. Results were expressed as a mean percentage of the GFP positive cells to the total cell number in each batch.

For nuclear import study of the specific *Xenopus* Lamin B3, cells cultivated on a glass coverslip were fixed in 100% methanol (30 min at −20 °C), rehydrated and incubated for 1 h at RT in PBS with 2% fraction V BSA. We observed that 4% PFA fixation induced non-specific immunostaining of the cells. Rabbit polyclonal primary antibody against *Xenopus* Lamin B3 (gift from N. Morin, France) diluted (1:4000) in PBS with 2% BSA was incubated overnight at 4 °C. Antibody specificity was validated on *Xenopus* stage 8 embryos (Supplementary Method 2). After washing with 0.2% BSA in PBS, the coverslip was incubated with goat anti-rabbit IgG Alexa Fluor®488 secondary antibody (1:400) for 1 h at room temperature. After washing, the coverslips were counterstained with 10 µg/mL Hoechst 33243 for 10 min, rinsed in PBS, and mounted on slides with glycerol (50% in water). The number of cells presenting nuclear Lamin B3 labelling just after the treatment and 24 h post resealing was counted in three independent batches (600 and 190 counted cells respectively). It was expressed as a mean percentage of the Lamin B3 positive cells to the total number of counted cells in each batch.

### *In vitro* culture of cells for recovery after treatment

For plasma membrane resealing, permeabilized cells treated or not with *Xenopus* egg-extract were cultivated in growth L15 medium supplemented with 2 mM CaCl2 (resealing medium) for 2 h at 25 °C. The resealing medium was then carefully removed and replaced with growth L15 medium. Cells were maintained in culture for several days at 25 °C. The cell behaviour was investigated by phase-contrast microscopy after the 2 h resealing and 24 h post-resealing. Membrane recovery was assessed at 24 h post-resealing by cell exposure to PI. Cell proliferation was determined using the 5′-Bromo-2′-deoxy-Uridine Labelling and Detection kit I (Roche, 11 296 736 001). Briefly, egg-extract treated cells at 24 h post resealing were incubated in 10 µM BrdU for 30 h. After fixation in ethanol 70% (in 50 mM glycine buffer pH 2) at −20 °C, BrdU labelling of the cells was revealed according to the manufacturer’s instructions. The number of BrdU stained cells was obtained from the counting of at least 250 cells, counterstained with 10 µg/mL Hoechst 33243 and expressed as a percentage of the total cell number. The counting was carried on three cultures with three independent extract batches.

### Statistical analysis

Means were compared by the t-test using Excel software. A *P* value < 0.01 was considered significant.

## Supplementary information

Supplementary Fig. S1, Supplementary Fig. S2:, Supplementary Fig. S3, Supplementary Method 1, Supplementary Method 2

## References

[1] Ganier O (2011). Synergic reprogramming of mammalian cells by combined exposure to mitotic Xenopus egg extracts and transcription factors. Proc Natl Acad Sci USA.

[2] Yang X (2012). Xenopus egg extract treatment reduced global DNA methylation of donor cells and enhanced somatic cell nuclear transfer embryo development in pigs. BioResearch open access.

[3] Miyamoto K (2007). Reprogramming events of mammalian somatic cells induced by Xenopus laevis egg extracts. Mol Reprod Dev.

[4] Miyamoto K (2009). Cell-Free Extracts from Mammalian Oocytes Partially Induce Nuclear Reprogramming in Somatic Cells. Biology of Reproduction.

[5] Hansis C, Barreto G, Maltry N, Niehrs C (2004). Nuclear reprogramming by Xenopus egg extract of human somatic cells requires BRG1. Current Biology.

[6] Alberio R, Johnson AD, Stick R, Campbell KH (2005). Differential nuclear remodeling of mammalian somatic cells by Xenopus laevis oocyte and egg cytoplasm. Exp Cell Res.

[7] Miyamoto K (2008). Reversible Membrane Permeabilization of Mammalian Cells Treated with Digitonin and Its Use for Inducing Nuclear Reprogramming by Xenopus Egg Extracts. Cloning and Stem Cells.

[8] Liu Y (2011). Cell Colony Formation Induced by Xenopus Egg Extract as a Marker for Improvement of Cloned Blastocyst Formation in the Pig. Cellular Reprogramming.

[9] Liu Y (2012). Increased blastocyst formation of cloned porcine embryos produced with donor cells pre-treated with Xenopus egg extract and/or digitonin. Zygote.

[10] Tang SA (2009). Reprogramming donor cells with oocyte extracts improves *in vitro* development of nuclear transfer embryos. Animal Reproduction Science.

[11] Chenais N, Depince A, Le Bail P-Y, Labbe C (2014). Fin cell cryopreservation and fish reconstruction by nuclear transfer stand as promising technologies for preservation of finfish genetic resources. Aquaculture International.

[12] Martinez-Paramo S (2017). Cryobanking of aquatic species. Aquaculture.

[13] Siripattarapravat K, Pinmee B, Venta PJ, Chang CC, Cibelli JB (2009). Somatic cell nuclear transfer in zebrafish. Nat Methods.

[14] Lee KY, Huang H, Ju B, Yang Z, Lin S (2002). Cloned zebrafish by nuclear transfer from long-term-cultured cells. Nature Biotechnology.

[15] Le Bail PY (2010). Optimization of somatic cell injection in the perspective of nuclear transfer in goldfish. BMC Dev Biol.

[16] Luo DJ, Hu W, Chen SP, Zhu ZY (2011). Critical developmental stages for the efficiency of somatic cell nuclear transfer in zebrafish. Int.J Biol Sci.

[17] Adam SA, Sternemarr R, Gerace L (1992). Nuclear-Protein Import Using Digitonin-Permeabilized Cells. Methods in Enzymology.

[18] Walev I (2001). Delivery of proteins into living cells by reversible membrane permeabilization with streptolysin-O. Proceedings of the National Academy of Sciences of the United States of America.

[19] Geelen MJH (2005). The use of digitonin-permeabilized mammalian cells for measuring enzyme activities in the course of studies on lipid metabolism. Analytical Biochemistry.

[20] Fiskum G, Craig SW, Decker GL, Lehninger AL (1980). The Cytoskeleton of Digitonin-Treated Rat Hepatocytes. P Natl Acad Sci-Biol.

[21] Adam SA, Marr RS, Gerace L (1990). Nuclear protein import in permeabilized mammalian cells requires soluble cytoplasmic factors. J Cell Biol.

[22] Liu Y (2014). Long-term effect on *in vitro* cloning efficiency after treatment of somatic cells with Xenopus egg extract in the pig. Reprod Fertil Dev.

[23] Chenais N, Lareyre JJ, Le Bail PY, Labbe C (2015). Stabilization of gene expression and cell morphology after explant recycling during fin explant culture in goldfish. Exp Cell Res.

[24] Adam SA, Sengupta K, Goldman RD (2008). Regulation of nuclear lamin polymerization by importin alpha. Journal of Biological Chemistry.

[25] Newport JW, Wilson KL, Dunphy WG (1990). A Lamin-Independent Pathway for Nuclear-Envelope Assembly. Journal of Cell Biology.

[26] Stick R (1988). cDNA Cloning of the Developmentally Regulated Lamin Liii of Xenopus-Laevis. Embo Journal.

[27] Rathbone AJ, Fisher PA, Lee JH, Craigon J, Campbell KHS (2010). Reprogramming of Ovine Somatic Cells with Xenopus laevis Oocyte Extract Prior to SCNT Improves Live Birth Rate. Cellular Reprogramming.

[28] Labbe C, Bussiere JF, Guillouet P, Leboeuf B, Magistrini M (2001). Cholesterol/phospholipid ratio in sperm of several domestic species does not directly predict sperm fitness for cryopreservation. Genetics Selection Evolution.

[29] Gorlich D, Kutay U (1999). Transport between the cell nucleus and the cytoplasm. Annual Review of Cell and Developmental Biology.

[30] Parisis N (2017). Initiation of DNA replication requires actin dynamics and formin activity. Embo Journal.

[31] Tramontina F (2000). Digitonin-permeabilization of astrocytes in culture monitored by trypan blue exclusion and loss of S100B by ELISA. Brain Res Brain Res Protoc.

[32] Adam SA, Gerace L (1991). Cytosolic Proteins That Specifically Bind Nuclear Location Signals Are Receptors for Nuclear Import. Cell.

[33] Kose S, Funakoshi T, Imamoto N (2015). Reconstitution of Nucleocytoplasmic Transport Using Digitonin-Permeabilized Cells. Nuclear Bodies and Noncoding Rnas: Methods and Protocols.

[34] Fennell DF, Whatley RE, Mcintyre TM, Prescott SM, Zimmerman GA (1991). Endothelial-Cells Reestablish Functional Integrity after Reversible Permeabilization. Arterioscler Thromb.

[35] Idone V (2008). Repair of injured plasma membrane by rapid Ca2+−dependent endocytosis. Journal of Cell Biology.

[36] Idone V, Tam C, Andrews NW (2008). Two-way traffic on the road to plasma membrane repair. Trends in Cell Biology.

[37] Mackay DJG, Esch F, Furthmayr H, Hall A (1997). Rho- and Rac-dependent assembly of focal adhesion complexes and actin filaments in permeabilized fibroblasts: An essential role for ezrin/radixin/moesin proteins. Journal of Cell Biology.

