# Supplementary material for: Nuclear import of Xenopus egg extract components into cultured cells for reprogramming purposes: a case study on goldfish fin cells
Source: Sci Rep. 2019 Feb 27;9:2861. doi: 10.1038/s41598-019-39500-y (PMC6393519; doi:10.1038/s41598-019-39500-y)

**Nuclear import of *Xenopus* egg extract components into cultured cells for reprogramming purposes: a case study on goldfish fin cells**

Nathalie Chênais*, Thierry Lorca, Nathalie Morin, Brigitte Guillet, Helene Rime, Pierre-Yves Le Bail, Catherine Labbé

***CORRESPONDING AUTHOR**
Nathalie Chênais: nathalie.chenais@inra.fr

**Supplementary Fig. S1 DNA fragmentation of the permeabilized cells maintained in culture without *Xenopus* egg extract.**

Cells in suspension were permeabilized by 30 µg/mL digitonin (Dig30) for 2 min at 4°C and were then maintained at 25°C in the presence (+ Extract) or absence (- Extract) of *Xenopus* egg extract. After 60 min, cells were labelled with Hoechst 33243. Note the presence of nuclear fragmentation (arrows) when the extract was absent. Scale bar = 20 µm.

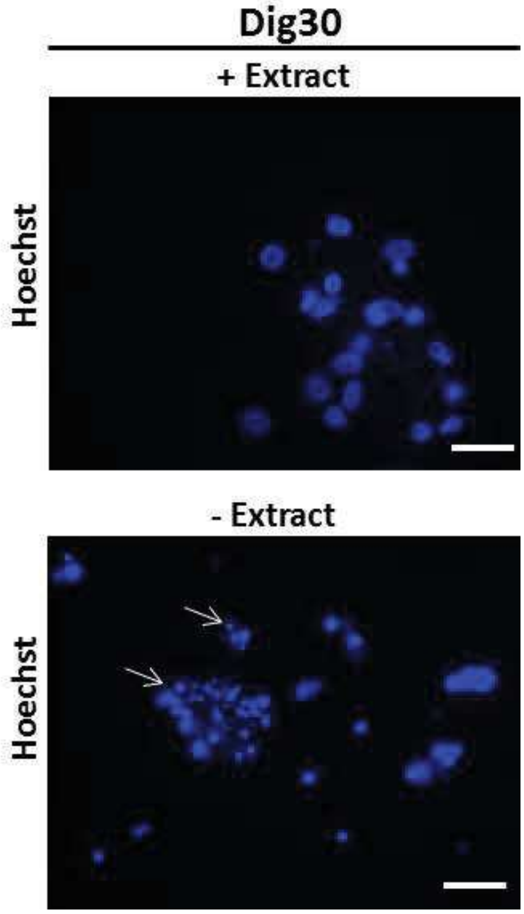

**Supplementary Fig. S2 Morphology of permeabilized cells treated with *Xenopus* egg-extract**

The morphology of permeabilized egg-extract treated (Dig30 + Extract) and non-permeabilized (Control) cells was observed by phase-contrast microscopy. Note that the cytoplasm is no longer distinguishable in treated cells. Only the nuclei remained strongly visible with a white appearance (arrows). Inset: magnification of one nucleus showing nucleoli very dark.

Scale bar = 20 µm.

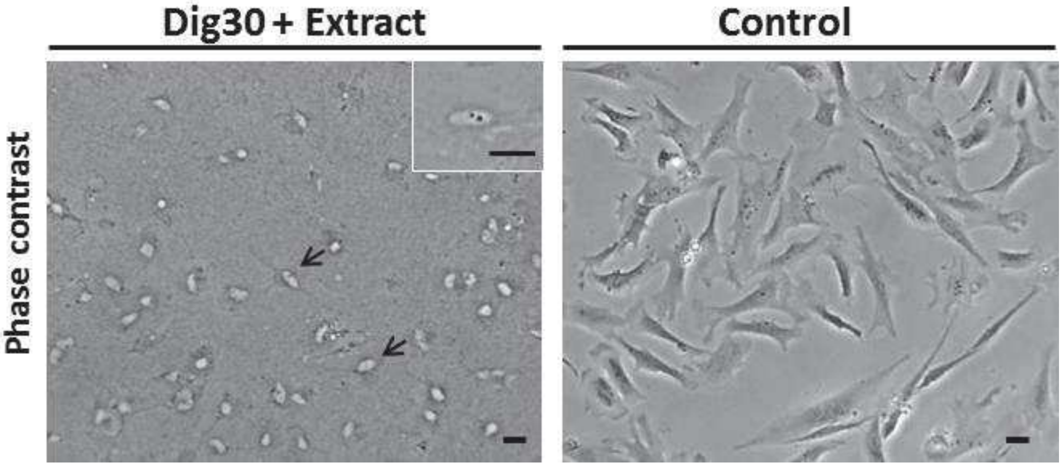

**Supplementary Fig. S3 Successful plasma membrane recovery of extract-treated permeabilized cells.**

Cells permeabilized with 30 µg/mL digitonin and treated with *Xenopus* egg-extract were incubated in resealing medium containing 2mM CaCl2 for 2 h at 25°C. After 24h, the cells were labelled with the non membrane permeant propidium iodide (PI) and Hoechst 33243 according the labelling condition described in Fig 2. No fluorescent PI signal was detected, indicating a successful plasma membrane recovery of the treated cells. These pictures are representative of 3 independent cultures in which 3 different egg extract batches were used.

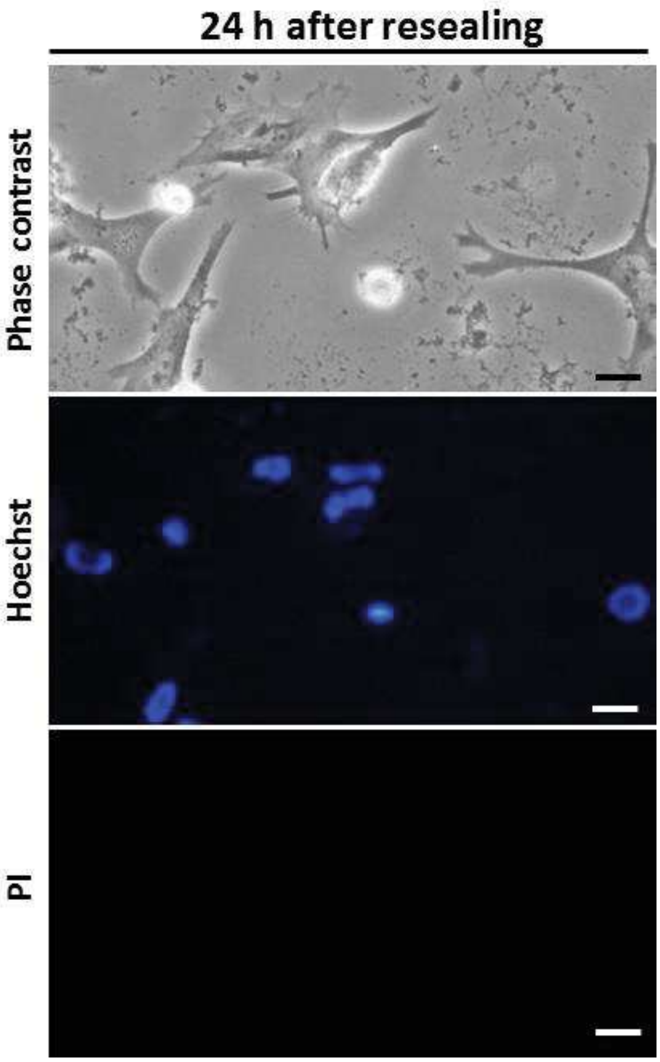

**Nuclear import of *Xenopus* egg extract components into cultured cells for reprogramming purposes: a case study on goldfish fin cells**

Nathalie Chênais*, Thierry Lorca, Nathalie Morin, Brigitte Guillet, Helene Rime, Pierre-Yves Le Bail, Catherine Labbé

***CORRESPONDING AUTHOR**
Nathalie Chênais: nathalie.chenais@inra.fr

## Supplementary Method 1. Fin primary cell culture conditions and egg extract preparation

Whole caudal fins were wiped to remove mucus, treated for 10 s in 70% ethanol and washed in PBS containing 100 µg /mL gentamycin and 2.5 µg/mL amphoterincin B. The whole fin was then minced and digested for 30 min with 2 mg/mL collagenase (Sigma C2674) in Leibovitz's L15 culture medium (Sigma L5520) supplemented with 5 mM Hepes, 2 mM NaHCO3 2 mM, 100 µg/mL gentamycin, 2.5 µg/mL amphotericin B (osmolality 290 mOsm/kg, pH 7.3) and 10% fetal calf serum, 2mM L-glutamine, 1% non-essential amino acids and 1% sodium pyruvate. After washing at 150 g for 10 min, the pellet was suspended in the culture medium and cells were cultured into six-well plates at 25°C. About 1.4 g fin provided enough material for one six well plate. An average of 8.5 ± 2.9 $10^6$ cells were recovered from an initial 1 g fin piece.

Unfertilized eggs from *Xenopus laevis* adult females (2-3 years old) were obtained after hCG stimulation from CRB xénopes (University of Rennes 1, agreement number: 35–238-42). Females were maintained in 100 mM NaCl during egg laying to preserve egg quality. The egg's jelly coat was removed with 2% cysteine solution (Sigma C7880) in 100 mM NaCl at pH 7.8 and eggs were washed with extraction buffer (200 mM KCl, 0.2 mM CaCl2, anhydrous 2 mM MgCl2, 20 mM Hepes, 100 mM sucrose and 12 mM EGTA, pH 7.8). Eggs were then packed by a short spin (400 x g) at 4°C. Extraction buffer was removed, and the eggs were crushed 10,600 x g for 20 min at 4°C. The extract was then clarified at 10,600 x g for 20 min at 4°C. The supernatant was collected, snap frozen in liquid nitrogen and stored at -80°C. The protein concentration of the egg extracts from four different spawns ranged from 40 to 50 mg/mL, with an osmolality of 400 mOsm/kg.

For western blot analysis, a fraction of egg extract 25 µg protein was mixed with Laemmli buffer. After denaturation at 95°C for 5 min, samples were separated by SDS-polyacrylamide gel electrophoresis (10 % 200 V, 1 h 30). Proteins were transferred to nitrocellulose membrane (Hybond-C super; Amersham, Life Science). The membranes were blocked for 1 h at RT in TBS with 0.1 % Tween 20 (TBS-T) and 5% non-fat dry milk (incubation buffer), then incubated overnight at 4°C with rabbit polyclonal *Xenopus* anti-Lamin B3 (1:10 000, a gift from N. Morin, France), *Xenopus* anti-importin alpha1 (1:5000, a gift from K. Weiss, Switzerland), mouse monoclonal rat anti-karyopherin β1 (1:1000 KPNB1, Antibodies-on-line, clone 23), and anti-beta actin (1:5000, Sigma, clone AC15). After washing, membranes were incubated 1 h at RT with anti-rabbit or anti-mouse secondary antibody conjugated to horseradish peroxidase (1:15 000 Jackson Immunoresearch). Immunoreactive bands were detected using Uptima Uptilight chemiluminescent revealent kit (Uptima-Interchim). Images were acquired with Fusion FX7 (Vilbert Lourmat).

## Supplementary Method 2. Specificity of the primary *Xenopus* Lamin B3 antibody: immunofluorescence labelling in *Xenopus* stage 8 embryos.

Whole *Xenopus* embryos were fixed with 100% methanol at -20°C overnight before paraffin embedding. Transversal sections (7 µm) of the animal pole were made and analyzed by immunofluorescence using the rabbit polyclonal primary antibody anti-*Xenopus* Lamin B3. Immunofluorescence and phase-contrast images

showed specific Lamin B3 labelling in the nuclei of blastomeres in the presence of antibody (+ AB). No significant signal was detected in embryo control section when primary antibody was omitted (-AB). Nuclei were counterstained using Hoechst 33243. Scale bar = 20 µm

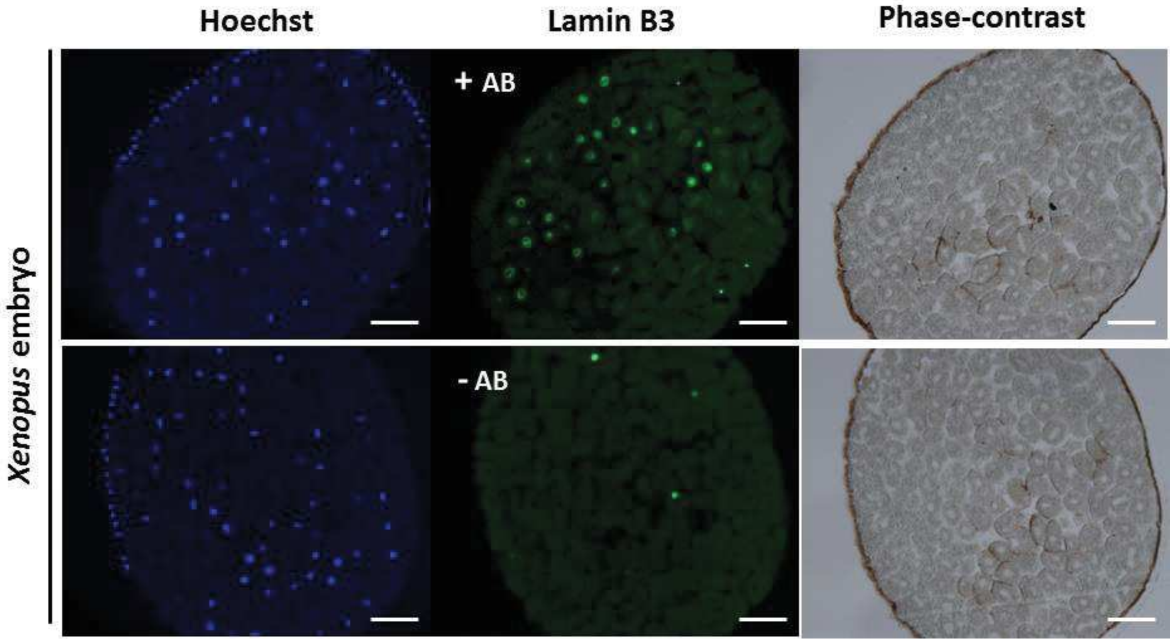

Supplement: Supplementary file 1 — Supplementary Fig. S1, Supplementary Fig. S2:, Supplementary Fig. S3, Supplementary Method 1, Supplementary Method 2 [file 41598_2019_39500_MOESM1_ESM.pdf]
